# Performance analysis method for model-based irrigation strategies under uncertainty

**DOI:** 10.1016/j.mex.2020.101075

**Published:** 2020-09-26

**Authors:** F.D. Mondaca-Duarte, M. Heinen, S. van Mourik

**Affiliations:** a Wageningen University, Farm Technology Group, P.O. Box 16, 6700 AH Wageningen, the Netherlands; b Wageningen Environmental Research, Wageningen UR, P.O. Box 47, 6700 AA Wageningen, the Netherlands

**Keywords:** Monte Carlo, Drainage, Richards equation

## Abstract

There is a necessity to increase the performance of food production in agriculture, this means, that precise management support in farming systems is required to reduce water use and drainage while avoiding crop stress. Management support based on model predictions is used to increase the performance of food production. However, sources of uncertainty affect the model predictions. Uncertainty in soil properties and uncertain evapotranspiration translate into uncertain predictions, and consequently in risk of performance loss. This paper presents the code and method to analyze performance uncertainty (and risk of performance loss) due to uncertain circumstances. The method is based on using the De Graaf evapotranspiration model and the EMMAN3G model, a Richards equation-based soil water model, as modules to conduct a performance uncertainty study.

## Highlights

• A Richards equation-based soil water model was combined with an evapotranspiration model to compute drainage and crop water stress resulting from irrigation within a MATLAB programming environment.
• Monte Carlo sampling was used to simulate how uncertainty in soil parameters and evapotranspiration propagates into predictions of drainage, water use and crop water stress.
• Soil water pressure head was used as a threshold value to indicate crop water stress based on soil profile interactions with the crop root zone.

## Graphical abstract

**Figure:**
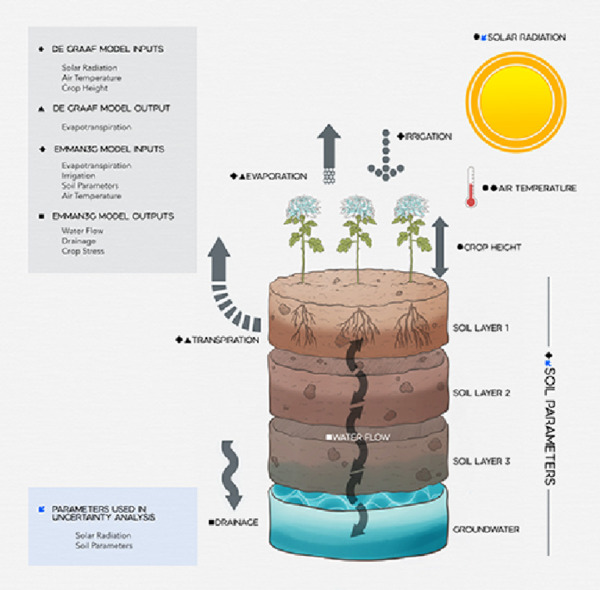

Specifications Table

**Table:** 

Subject Area	Agricultural and Biological Sciences
More specific subject area	*Model-Based irrigation, Precision Farming, uncertainty analysis*
Method name	*Uncertainty framework for model-based irrigation*
Name and reference of original method	
Resource availability	

## Method details

In this contribution we present the code used in [3] to study the impact of uncertainty in evapotranspiration and soil parameters on drainage and crop stress predictions. For that, we adopted a model that mimics the real world in terms of the simulation model that describes water movement in soil (the EMMAN3G model). The software package (in Fortran) of the EMMAN3G, as described in [2], is included. Details of the model can be found in [3] and are briefly repeated here.

Based on the uncertainty in model input and model parameters, a Monte Carlo analysis was developed in order to compute the uncertainty in predictions. This framework was developed in a MATLAB 2018b environment.

## Main concepts

### Richards equation

The Richards equation describes the change of water content in a soil column based on the water pressure head, hydraulic conductivity, water uptake, and irrigation:

(1) $$\frac{\partial \theta }{dt}=\frac{\partial }{\partial z}\left(K\left(\theta \right)\frac{\partial h\left(\theta \right)}{\partial z}\right)-\frac{\partial K\left(\theta \right)}{\partial z}-Sr.\;\;\;\left[{\text{ml}\;\text{cm}}^{-3}\;d^{-1}\right]$$

Here *θ* is the volumetric water content [ml cm^−3^], *t* is the time, *z* is the vertical coordinate oriented positive downwards [cm], *K* is the hydraulic conductivity [cm d^−1^], *h* is the pressure head [cm], and *Sr* is a sink term accounting for crop water uptake, uniformly distributed over the crop area and uniformly distributed within the first 30 cm of soil layer [ml cm^−3^ d^−1^]. Eq. (1) is non-linear due to the non-linear constitutive relationships between *h, θ*, and *K*, as presented next, and thus needs to be solved numerically.

### The Mualem-Van Genuchten relationship

The Van Genuchten function (Eq. (2); [1]) describes the water retention as a function of the pressure head according to

(2) $$S\left(h\right)=\frac{\theta \left(h\right)-\theta_{r}}{\theta_{s}-\theta_{r}}=\left\{ \begin{array}{cc} \frac{1}{{\left(1+{\left|\alpha h\right|}^{n}\right)}^{m}} & h\leq 0\\ 1 & h>0 \end{array}\right..\;\;\;\left[-\right]$$

Here *S* is the effective saturation [dimensionless], *θ_r_* is the residual water content, *θ_s_* is the water content at saturation, and *α* [cm^−1^], *n* [dimensionless], and *m* [dimensionless] are curve shape parameters. The hydraulic conductivity characteristic is given by the Mualem equation (Eq. (3)) [4] (with m=1−1/n)

(3) $$K_{r}\left(S\right)=\frac{K\left(S\right)}{K_{s}}=S^{\lambda }{\left[1-{\left(1-S^{\frac{1}{m}}\right)}^{m}\right]}^{2}.\;\;\;\left[-\right]$$

Here *K_r_* is the relative hydraulic conductivity [dimensionless], *K_s_* is equal to *K* at saturation [cm d^−1^], and *λ* is a curve shape parameter [dimensionless].

### Root water uptake

Under well-watered conditions it is known that root water uptake is proportional to the root length density distribution. Since the main aim of crop production in greenhouse conditions is to maintain optimal conditions in the root zone, root water uptake was assumed to be proportional to fractional root distribution in the root zone times the potential transpiration. When this yields a pressure head exceeding a certain threshold value, this is regarded as a signal of water limitation.

### The De Graaf model

The De Graaf model (Eq. (4); [5]) is used to calculate the evapotranspiration inside a greenhouse based on the global radiation received, the additional radiation by artificial lighting, the air temperature inside the greenhouse, the additional air temperature supplied by heating pipes, and the ratio between actual crop length and maximum crop length. This is formulated as follows,

(4) $$ET=\left(aR+b|T_{tube}-T_{gh}|\right)\frac{L}{L_{max}}.\;\;\;\left[\text{mm}\right]$$

Here *ET* [mm] is the evapotranspiration, *R* [J cm^−2^] is the global radiation outside the greenhouse combined with the radiation from supplementary light, *T_tube_* [°C] is the temperature from heating pipes, *T_gh_* [°C] is the greenhouse indoor air temperature, *a* [mm cm^2^ J^−1^] is an empirical crop factor for the effect of radiation, *b* [mm °C^−1^] is an empirical crop factor of the heating influence, *L* is the current crop length, and *L_max_* is the maximum crop length. In this study we focused on the final stage of the cropping period so that $L=\;L_{max}$.

The *ET* is then divided in the potential transpiration (demand for crop water uptake) *T*_pot_ and potential evaporation at the soil surface *E*_pot_ according to

(5) $$T_{pot}=\;ET\left(1-e^{\left(-0.6*LAI\right)}\right).\;\;\;\left[\text{mm}\right]$$

(6) $$E_{pot}=\;ET-T_{pot}.\;\;\;\left[\text{mm}\right]$$

where *LAI* is the leaf area index. For a nearly full grown crop, as considered here, *ET* is dominantly assigned to *T_pot_*, and, therefore, we used $T_{pot}=\;ET$ and $E_{pot}=0$.

## MATLAB framework code

### The De Graaf model

First, the De Graaf model [5] was set up as a function



### Starting the model framework and data input

A script is used to start the framework. First, any stored previous data is cleared and data sets of irrigation, temperature, and radiation are stored as single column double arrays. If multiple irrigation strategies are used then for each irrigation, the values are stored in a separate column, under *irrigation.mat*



Next, the evapotranspiration function is run and stored as the variable *ET*



### Simulation settings

An IF statement was used to select if the simulations will include uncertainty or not. The variables *et_unc* and *soil_unc* indicate if uncertainty will be present in the study (>0) or not (= 0). After, the number of days the dataset spans are included. Next, the possible clay and sandy soils names were included in cell values named *clay_soils* and *sand_soils*. If evapotranspiration is uncertain, the evapotranspiration data is divided into the total number of days the dataset represents and stored as the variable *ET_unc*. Depending on the type of soil, an IF statement stores the clay or sand type of soil as the variable t*ype_soil.*



After selecting whether soil or evapotranspiration uncertainty will be included, the file *PlantToSoil.dat* is read as a ASCII delimited file. Then, the number of different irrigation strategies to run, and the number of sampling repeats using Monte Carlo sampling, are established as variables.



### Monte Carlo sampling – evapotranspiration uncertainty

A FOR loop of the number of irrigation strategies is included as *irrigations_run*. Within the *irrigations_run* loop, another FOR loop of the number of times the framework will run depending on the number of Monte Carlo samplings as *model_run*. Within the *model_run* FOR loop, an IF statement and a FOR statement are added to indicate the Monte Carlo sampling of ET values. The FOR loop *ET_unc_run* includes a random sampling of *ET_unc* storing them as the variable *ET_sampling* to create a new *ET* variable for the current run simulation.



### Monte Carlo sampling – soil uncertainty

An IF statement is added to indicate if soil uncertainty is included in the study when the variable *soil_unc* is higher than zero. Within the IF statement, a Monte Carlo random sampling of the soil parameters is included from the soil list *type_soil* and stored as the variable *soil*. The *SoilInfo.dat* is opened using the ‘fopen’ command and giving the ID *fid_soil*. Data from *fid_soil* is read using the command ‘textscan’ and stored as a string variable *soil_data. Fid_soil* is closed using the ‘fclose’ command, and the *soilinfo.dat* file is re-opened using the ‘fopen’ command with the option to rewrite. The new soil values stored in the variable *soil_data* are written into *SoilInfo.dat* using the ‘fprintf’ command. Finally *fid_soil* is closed using the ‘fclose’ command.



### Sending the case studies to the EMMAN3G model

New values for evapotranspiration, temperature and irrigation are written on the previous established variable *plant_soil*. Evaporation and root length are commented out as it was assumed in the study [3] that the crop was fully grown, so evaporation from soil was neglected, and root length was assumed to be a constant of 30 cm. The file *PlantToSoil.dat* is re-opened with writing rights using the ‘fopen’ command. The headers for the data are included with the ‘fprintf’ command. The data from *plant_soil* is written to *PlantToSoil.dat* using the command ‘dlmwrite’ (ASCII delimited file) with a specific precision required by the EMMAN3G model and using ‘-append’ to append the matrix to the file, if not, ‘dlmwrite’ overwrites the previous included headers.



### Running the EMMAN3G model

Before running the EMMAN3G model, the previous output file is deleted. EMMAN3G model is run using the ‘system’ command. A new *EmMan3G_W.csv* file with be created by the EMMAN3G model.



### Gathering results data

The *EMMAN3G_W.csv* file is opened and the number of columns are established as the variable *columns_number*. This is because, depending on the number of Z planes in the *LocalFlux.dat* file, the number of columns of the csv file will change. The data in the csv file is saved in the variable *csv_data* using the ‘textscan’ command. The fid is closed and the data is reshaped into the number of original rows and columns, and is stored in the variable *data*.



### Storing results data as MATLAB variables

The variable *data* is in strings format and it is required to be as double. A FOR loop is used to store the results from the data variable as cells for the results of drainage, water pressure head of the first soil layer (8 Z-planes) and crop transpiration.



Each Monte Carlo sampling iteration outputs values of drainage, pressure head and transpiration predictions. The values stored in *drainage, pressure_head*, and *transpiration* are stored in new variables with the suffix _MC, which changes in which column the data is stored depending on the FOR loop *model_run*. Finally, the FOR loop *model_run* is closed after completing the Monte Carlo samplings.



Finally, the Monte Carlo sampling results for drainage, crop transpiration and water pressure head are stored in a cell which changes cell position depending on the irrigation strategy FOR loop *irrigations_run*. The FOR loop *irrigations_run* is closed after completing the different irrigation strategies previously established.



### Defining crop stress ratio

The crop stress ratio consists of values of the mean water pressure head that are lower than the selected variable *threshold.* The crop is considered to be under stress when the mean water pressure is below the threshold. Three nested FOR loops were used to run the crop water stress ratio. The first loop runs for the number of irrigation strategies established (*stress_irrigation_run*). The next loop runs for the number of Monte Carlo samples (*stress_MC*). The third FOR loop runs for the number of water pressure head values stored in *final_pressure_head* variable.

The mean pressure head value is obtained from the mean of the bottom 4 Z planes in the first soil layer. The mean value is stored in the variable *mean_p_head*. Also, the variable mean_p_head stores the mean pressure head value from different irrigation strategies studied in separate rows of the variable. If the values of *mean_p_head* are below the *threshold* value then it is stored as a risk event under the variable *risk*. The crop stress ratio represents the ratio of hours the crop was below the threshold value over the total amount of hours. The ratio values are stored in a cell (*final_risk*), this cell changes cell positions to store the ratio values of the different irrigation strategies within the FOR loop *stress_irrigation_run.*



### Display of results

The mean and standard deviation values of the model outputs: drainage, risk of crop stress, and evapotranspiration was represented graphically. This was done with the FOR loop *plot_results*, storing the mean and standard deviation results in variables with the first column as the mean and the second column as the standard deviation. See Table 1 for a tabulated data example.

**Table 1:** Output tabulated data example. Mean and standard deviation values are stored in three variables.

plot_risk		plot_drainage		plot_ET	
mean	std	mean	std	mean	std
0.66	0.14	1.54	1.05	114.6	6.2
0.45	0.18	3.06	2.51	115.5	6.0
0.25	0.15	6.80	3.85	115.1	6.2
0.12	0.10	14.35	5.23	114.0	6.2
0.08	0.07	21.23	5.29	115.8	5.8
0.03	0.04	29.87	5.67	114.7	6.1
0.03	0.04	38.40	5.92	114.9	6.1
0.02	0.03	47.03	6.11	115.6	6.1



**Fig. 1:**
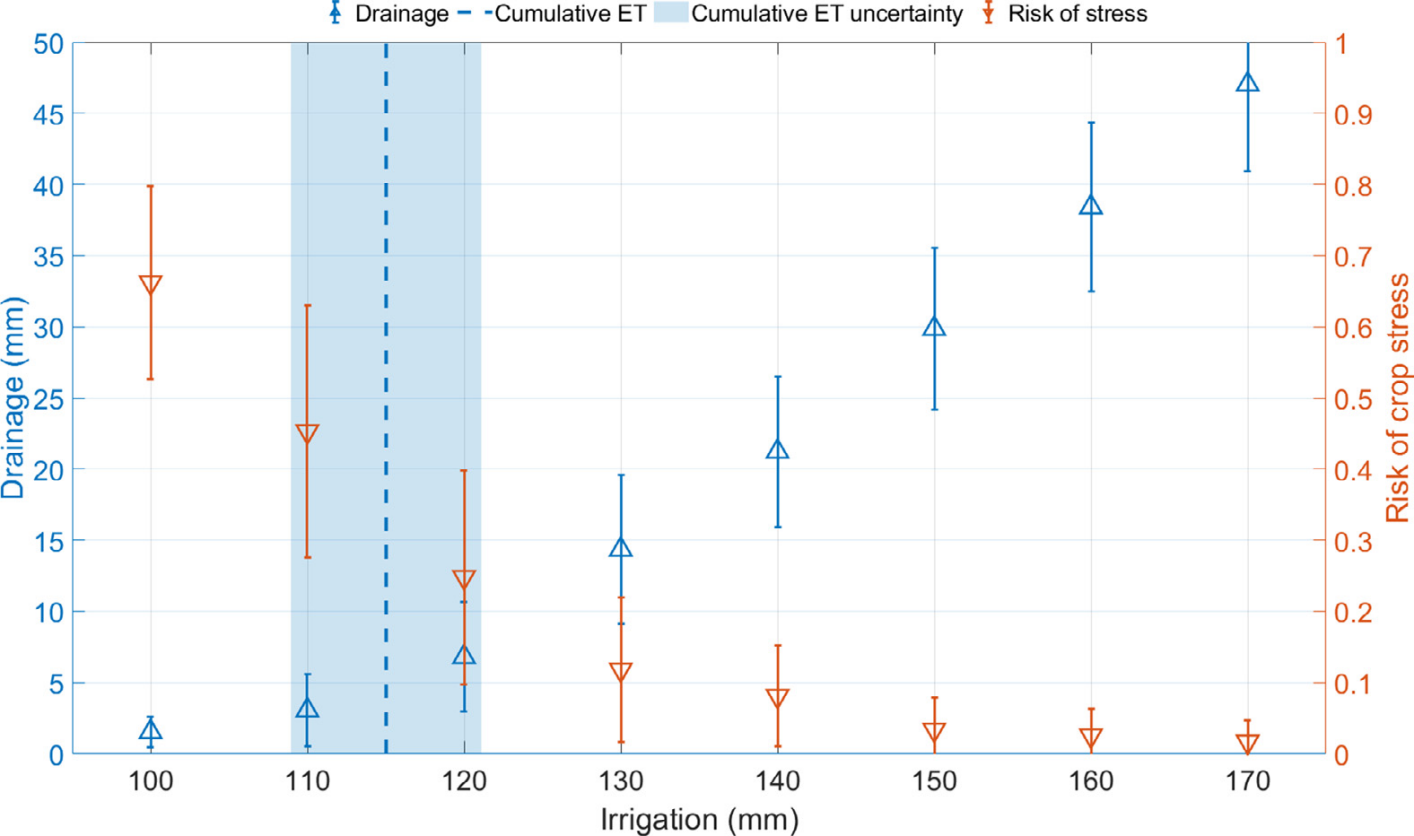
Example of a case study using the tabulated data. The shadowed area represents the uncertainty in the cumulative evapotranspiration. The bars represent the standard deviation of the prediction due to uncertainty in evapotranspiration.



A figure is created, the X-axis is established by including a variable (*total_irrigation*) which includes the different irrigation values used as irrigation strategies. The command ‘Yyaxis right’ is used to set an errorbar figure for the risk, using the *plot_risk* variable first column for the mean and the second column for the error. ‘Yyaxis left’ is used to set another errorbar for the drainage, using *plot_drainage* variable first column for the mean and the second column for the error. The uncertainty in evapotranspiration was represented as a set of limits between the mean and standard deviation of the *plot_ET* variable stored as the variable *rx1* for the lower limit and variable *rx2* for the higher limit. The space between *rx1* and *rx2* was filled an colored using the ‘area’ command. This colored area was made transparent using the ‘FaceAlpha’ and removing the line area with ‘LineStyle’. See Fig. 1 as an example of the figure created using the tabulated data.

## Declaration of Competing Interest

The authors declare that they have no known competing financial interests or personal relationships that could have appeared to influence the work reported in this paper.
